# Change in the plasma proteome associated with canine cognitive dysfunction syndrome (CCDS) in Thailand

**DOI:** 10.1186/s12917-021-02744-w

**Published:** 2021-01-29

**Authors:** Sataporn Phochantachinda, Boonrat Chantong, Onrapak Reamtong, Duangthip Chatchaisak

**Affiliations:** 1 Faculty of Veterinary Science, Mahidol University, Salaya, Phutthamonthon, Nakorn Pathom 73170 Thailand; 2 Department of Pre-Clinical and Applied Animal Science, Faculty of Veterinary Science, Mahidol University, Salaya, Phutthamonthon, Nakorn Pathom 73170 Thailand; 3 Department of Molecular Tropical Medicine and Genetics, Faculty of Tropical Medicine, Mahidol University, Phaya Thai, Ratchathewi, Bangkok 10400 Thailand; 4 Department of Clinical Sciences and Public Health, Faculty of Veterinary Science, Mahidol University, Salaya, Phutthamonthon, Nakorn Pathom 73170 Thailand

**Keywords:** Amyloid beta 42, Canine cognitive dysfunction syndrome, LC-MS/MS

## Abstract

**Background:**

Canine cognitive dysfunction syndrome (CCDS) is a progressive neurodegenerative disorder found in senior dogs. Due to the lack of biological markers, CCDS is commonly underdiagnosed. The aim of this study was to identify potential plasma biomarkers using proteomics techniques and to increase our understanding of the pathogenic mechanism of the disease. Plasma amyloid beta 42 (Aβ_42_) has been seen to be a controversial biomarker for CCDS. Proteomics analysis was performed for protein identification and quantification.

**Results:**

Within CCDS, ageing, and adult dogs, 87 proteins were identified specific to *Canis* spp*.* in the plasma samples. Of 87 proteins, 48 and 41 proteins were changed in the ageing and adult groups, respectively. Several distinctly expressed plasma proteins identified in CCDS were involved in complement and coagulation cascades and the apolipoprotein metabolism pathway. Plasma Aβ_42_ levels considerably overlapped within the CCDS and ageing groups. In the adult group, the Aβ_42_ level was low compared with that in the other groups. Nevertheless, plasma Aβ_42_ did not show a correlation with the Canine Cognitive Dysfunction Rating scale (CCDR) score in the CCDS group (*p* = 0.131, R^2^ = 0.261).

**Conclusions:**

Our present findings suggest that plasma Aβ_42_ does not show potential for use as a diagnostic biomarker in CCDS. The nano-LC-MS/MS data revealed that the predictive underlying mechanism of CCDS was the co-occurrence of inflammation-mediated acute phase response proteins and complement and coagulation cascades that partly functioned by apolipoproteins and lipid metabolism. Some of the differentially expressed proteins may serve as potential predictor biomarkers along with Aβ_42_ in plasma for improved CCDS diagnosis. Further study in larger population-based cohort study is required in validation to define the correlation between protein expression and the pathogenesis of CCDS.

**Supplementary Information:**

The online version contains supplementary material available at 10.1186/s12917-021-02744-w.

## Background

Canine cognitive dysfunction syndrome (CCDS) or ‘canine dementia’ is a neurodegenerative disease causing behavioural changes that are characterized by gradual reductions in learning, memory, spatial awareness, social interactions and sleeping patterns [1]. The prevalence of CCDS is high and affects up to 60% of mostly dogs older than 11 years [2]. There is no breed-specific difference in the clinical signs or pathology of the disease [3]. However, clinical signs of CCDS are more often observed and reported in smaller dogs [4, 5].

Several studies of neurodegenerative diseases in animals have shown strong similarities in pathology and characteristics between cognitive dysfunction in dogs and Alzheimer’s disease (AD) in humans [6–8]. Typical pathological hallmarks of CCDS are characterized by cortical atrophy, ventricular widening, demyelination, neuronal loss and the presence of amyloid beta (Aβ) plaques in the brain parenchyma and vessels such as AD in humans [9, 10]. The inflammation cascade and oxidative stress have been proposed to be underlying mechanisms of AD. An immunohistochemical study of the CCDS brain showed Aβ accumulation on meningeal vessels [11]. Aβ accumulation in the brain shows a significant relation with cognitive decline [9, 12]. In AD, Aβ plaque-activated glial cells were postulated as a putative mechanism for chronic inflammation [13]. Aβ deposition and diffuse plaque formation lead to microglial activation and many inflammation-related proteins, such as complement factors, acute phase proteins, chemokines and cytokines, such as interleukin-1β (IL-1β), interleukin-6 (IL-6), tumour necrosis factor-α (TNF-α), and transforming growth factor-β (TGF-β) [14–16]. Genetically, the *ε4* allele of the *apolipoprotein E* (*APOE*) gene has been identified as a main risk factor in late-onset AD [17]. Aβ clearance in the brain depends on the affinity between Aβ and the *APOE gene.* Moreover, the *APOE ε4* allele has low affinity for Aβ, affecting the clearance mechanism in AD [18]. Nevertheless, there were no reported specific genes involved in CCDS [19]. In the human brain, there are two main forms of Aβ (Aβ_40_ and Aβ_42_). The accumulation of Aβ_42_ is more toxic and is more related to the pathologies of AD than the accumulation of Aβ_40_ [20]. A correlation was observed between the severity of cognitive deficit in dogs and the density of Aβ_42_ accumulation in their brains [21].

Under normal conditions, Aβ is in equilibrium between biosynthesis and clearance. The clearance of Aβ from the brain can be completed by several mechanisms through nonenzymatic pathways, such as transport across vessel walls into blood circulation or enzymatic pathways, including neprilysin and insulin-degrading enzymes [22]. Cerebral Aβ is transported across the blood-brain vessel walls through scavenger receptors such as lipoprotein receptor-related protein 1 (LRP1) and very low-density lipoprotein receptor (VLDLR) into the blood circulation. Sequester proteins increase affinity to binding with scavenger receptors [20] and function as stabilizers of monomeric Aβ with the inhibition of Aβ aggregation [23]. Sequester proteins such as alpha-2-macroglobulin, apolipoprotein E (apo E) and transthyretin were shown to increase the capability to transport Aβ via LRP1 [24, 25]. The relation between sequester proteins and Aβ may serve as a diagnostic biomarker in human AD.

At present, CCDS diagnosis depends on observations from owners and veterinarians. No biological marker allows accurate CCDS diagnosis [19]. Screening questionnaires with a list of clinical signs have been used as diagnostic tools in the veterinary field. Several questionnaires have proposed criteria for the diagnosis and staging of CCDS [2, 21, 26–29]. The Canine Cognitive Dysfunction Rating scale (CCDR) is one of the most frequently used questionnaires with high diagnostic accuracy (98.9%) [27, 30, 31]. The CCDR includes assessment of behaviour frequency and the categorization of the score for identification as non-CCD, the risk of developing CCD, and CCD [27]. In Thailand, CCDS is also a major health problem in older dogs. The prevalence of Thai CCDS in dogs between 7 and 12 years old ranged from 43 to 68%, and the prevalence of CCDS increased with age [32]. Even though many candidate biomarkers have been identified in both blood and cerebrospinal fluid (CSF), none of these markers has been used routinely in the clinic. In previous studies, plasma Aβ_42_ was evaluated as a biomarker in AD and CCDS, but it is highly variable and seems to be controversial [7, 33, 34]. Therefore, the identification and characterization of novel biomarkers are necessary for the reliable diagnosis of CCDS.

Proteomics is one of the most significant techniques that allows an extended investigation of neurodegenerative diseases. A blood-based proteomics approach was used extensively in humans with AD to study potential biomarkers or mechanisms related to this disease [35–37]. Proteomics in AD revealed many interesting proteins or pathways from CSF and blood, such as fatty acid oxidation and the advanced glycation end products/receptors for advanced glycation end products (AGE/RAGE) pathway [38, 39]. Moreover, there are no reports of serum proteome profiles in CCDS, and blood-based proteomics approaches in dogs are limited.

In the present study, we determined the association between the plasma Aβ_42_ expression level by the ELISA technique in adult, ageing and CCDS dogs and the CCDR score. Proteomics techniques were used to identify a dataset of potential plasma biomarkers and to investigate the underlying mechanisms of CCDS. These findings may provide new insights into the underlying mechanisms of CCDS. Moreover, potential plasma biomarkers from LC-MS/MS may be helpful and applied together with questionnaires in the evaluation of CCDS.

## Results

### Population characteristics

The baseline characteristics of dogs in proteomic study are shown in Table 1. There were no significant differences in sex, weight, and breed among the three groups. The haematological and blood chemistry which evaluated kidney and liver function were not significantly different between the three groups**.** The protein in plasma was also not significantly different between the three groups and within each group.

**Table 1:** The baseline characteristics of dogs include in proteomic study

Group	No	Age (years)	Sex	Weight (Kg)	Breed	WBC (× 10^**3**^)	Hct (%)	ALT (U/L)	Creatinine (mg/dL)	Protein (μg/μl)
CCDS	1	15	F	17	Mongrel	5.3	38	80	1.13	3.53
	2	13	F	11	Mongrel	16	41.9	195	0.84	3.57
	3	17	M	23	Mongrel	10.8	33.6	55	0.94	3.42
	4	12	F	20	Mongrel	12.1	39.2	95	1.33	3.55
Ageing	1	15	F	15	Mongrel	13.4	38	77	1.77	3.89
	2	12	M	25	Mongrel	6.9	39.4	179	1.52	3.92
	3	12	F	19	Mongrel	10.5	36	304	1.65	3.87
	4	12	F	30	Mongrel	10.2	43	236	1.23	3.92
Adult	1	3	M	14	Mongrel	14.2	36.5	22	1.25	4.04
	2	1	M	14	Mongrel	6.9	40	175	1.55	3.84
	3	3	M	13	Mongrel	13.7	34	26	1.6	4.01
	4	1	F	13	Mongrel	6.3	39	76	1.35	3.74

White blood cells (WBC); *p* = 0.9036, Haematocrit (Hct); *p* = 0.8126, Alanine aminotransferase (ALT); *p* = 0.0976, creatinine; *p* = 0.0548

### Correlation between the CCDR score and plasma Aβ_42_ levels

**Fig. 1:**
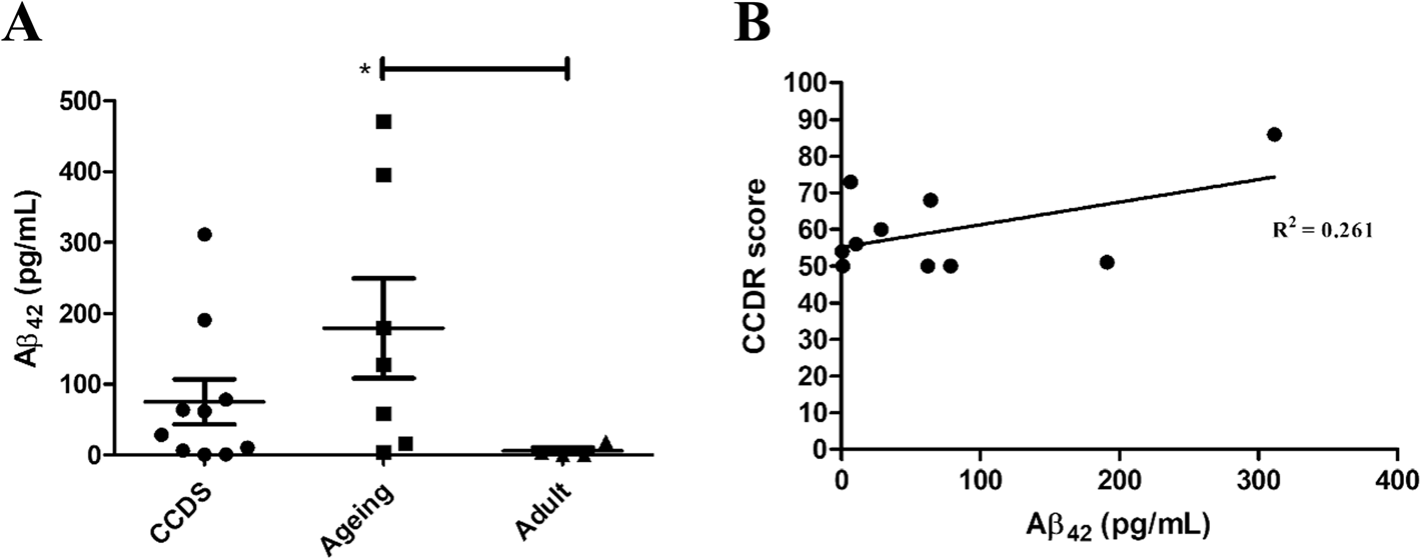
Plasma Aβ_42_ concentration **a.** Plasma Aβ_42_ concentrations (pg/μL) in each group (*p = 0.038) **b.** Plasma Aβ_42_ concentrations and CCDR scores in the CCDS group

First, levels of plasma Aβ_42_ in CCDS dogs (*n* = 10) were lower than those in ageing dogs (*n* = 7) but higher than those in the adult group (*n* = 4) (for Aβ_42_ ± SD: 75.40 ± 101 pg/mL in CCDS, 179.21 ± 185.6 pg/mL in the ageing group and 5.88 ± 9.28 pg/mL in the adult group). The Aβ_42_ level in the ageing and adult group was shown a significant difference (* *p* = 0.038) Nevertheless, there were no significant differences between CCDS with other groups. (Fig. 1 a). Furthermore, clinical diagnosis of the CCDS group was performed with the CCDR questionnaire. CCDR scores above 50 are indicative of CCDS in older companion dogs. Second, we evaluated individual plasma Aβ_42_ levels in all dogs. Our study showed that plasma Aβ_42_ levels in the CCDS group were not correlated with the CCDR score (*p* = 0.131, R^2^ = 0.261) (Fig. 1 b).

### Proteomics profile

**Fig. 2:**
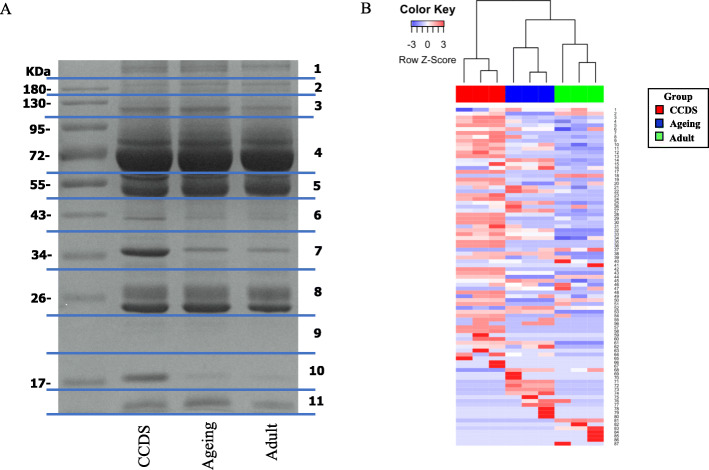
Protein patterns using **a**. SDS-PAGE with Coomassie blue R-250 staining (original gel image was in supplement information) **b**. Heat map of differentially expressed protein patterns

Plasma proteins of the CCDS group were compared with others by a proteomics approach to study differential protein expression. The proteins were separated by One-dimensional sodium dodecyl sulfate-polyacrylamide gel electrophoresis (SDS-PAGE). Each lane was cut into 11 pieces (Fig. 2a) prior to in-gel trypsin digestion. The digested peptides were identified by nano-LC-MS/MS analysis, and each protein was quantified using an exponentially modified protein abundance index (emPAI) value from the label-free spectral counting technique. In total, 1037 proteins were identified in the plasma samples from domestic dogs in Thailand against a non-redundant National Center for Biotechnology Information (NCBInr) database specific to *Mammalia* spp*.* as a taxonomic filter.

The protein bands in SDS-PAGE of CCDS samples in rows 5, 7 and 10 were different than those of the other groups by macroscopic appearance (Fig. 2 a). Data from nano-LC-MS/MS unveiled proteins in band 5 of CCDS samples that were composed of immunoglobulin gamma heavy chain B, fibrinogen beta chain, alpha-2-HS-glycoprotein, fibrinogen gamma chain, immunoglobulin gamma heavy chain C, vitamin D-binding protein, beta-2-glycoprotein 1 precursor and immunoglobulin A heavy chain constant region. The proteins in band 7 of CCDS samples were composed of haptoglobin, complement C4-A and immunoglobulin lambda-like polypeptide 5-like. The proteins in band 10 of CCDS samples were composed of haptoglobin.

**Fig. 3:**
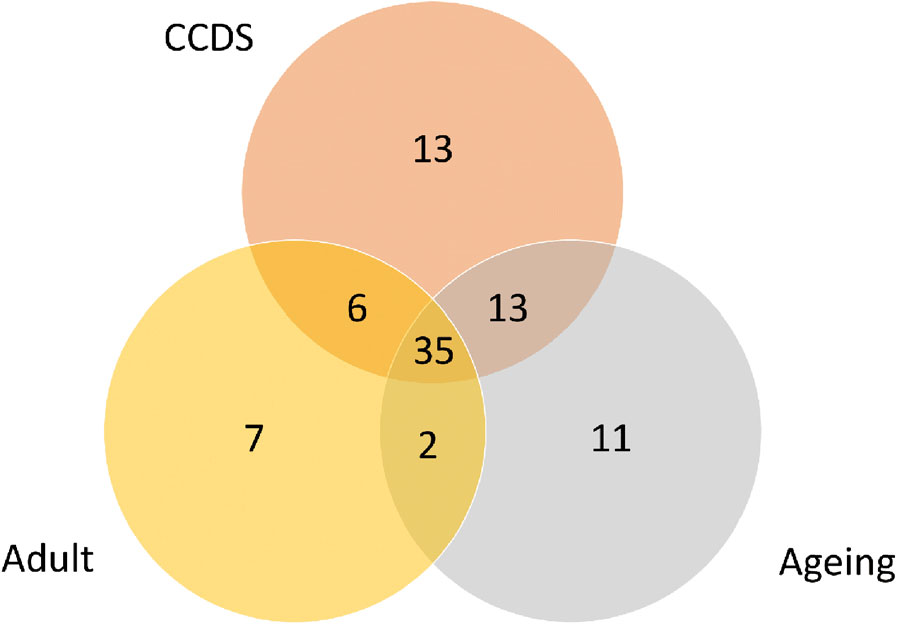
Venn diagram of protein detection (Canis spp.)

Heat map analysis, presenting differential capability of protein expression, illustrated 3 distinct groups (Fig. 2 b). For species specificity, proteomic data were identified only for *Canis* spp. A Venn diagram analysis shows the number of proteins overlapping between the three datasets. A total of 87 canine proteins were matched, and 35 (40.2%) of those were detected in all three groups. The number of proteins identified overlapping between CCDS and ageing group was 48 (55.1%) and number of proteins identified overlapping between CCDS and adult group was 41 (47.1%) (Fig. 3).

**Fig. 4:**
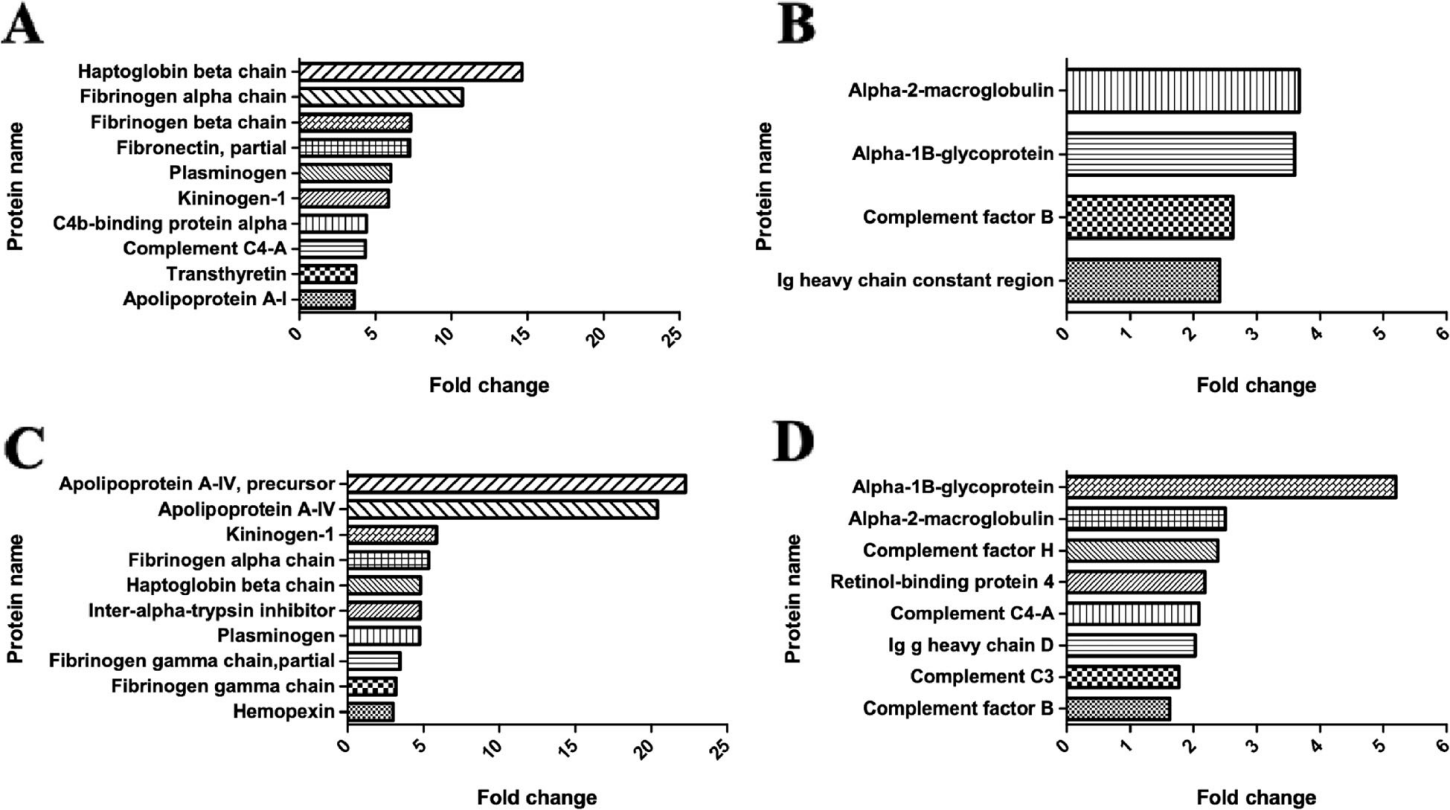
The changes in most proteins ranked by emPAI value: **a**. Upregulated proteins in comparisons between the CCDS group and the adult group **b**. Downregulated proteins in comparisons between the CCDS group and the adult group **c**. Upregulated proteins in comparisons between the CCDS group and the ageing group **d**. Downregulated proteins in comparisons between the CCDS group and the ageing group

The most upregulated proteins in the CCDS group compared with the adult group were involved with the coagulation cascade, while the most upregulated proteins in the CCDS group compared with the ageing group were involved with apolipoprotein. The most downregulated proteins in the CCDS group compared with the adult group or the ageing group were alpha-2-macroglobulin and alpha-1B-glycoprotein. Quantification was performed using emPAI provided by Mascot. The emPAI values in this report were the mean of three biological replications. A different expression of proteins at least two replicates were reported as altered proteins. In Fig. 4, the top ten most upregulated and downregulated proteins in comparisons between the CCDS group and the adult group are demonstrated in (a) and (b), respectively. Whereas, the top ten most upregulated and downregulated proteins in comparisons between the CCDS group and the ageing group are presented in (c) and (d), respectively.

### Gene ontology (GO) and pathway enrichment analyses of proteins

**Fig. 5:**
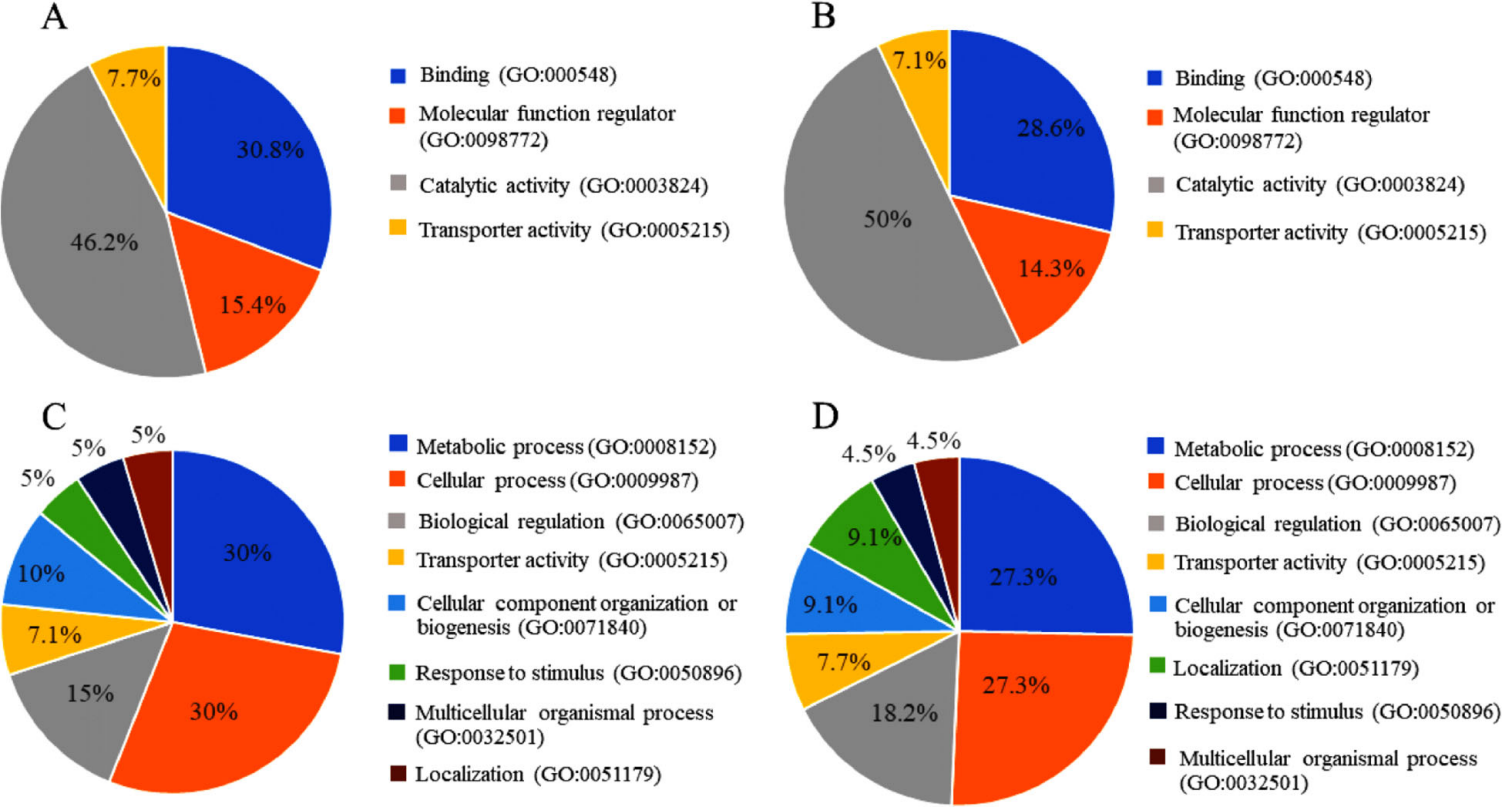
Gene Ontology molecular function and biological process categories for upregulated proteins in the CCDS group: **a**. comparison of the CCDS group with the ageing group in molecular function; **b**. comparison of the CCDS group with the adult group in molecular function; **c**. comparison of the CCDS group compared with the ageing group in biological process; and **d**. comparison of the CCDS group with the adult group in biological process

We first mapped the proteins onto GO databases via the PANTHER database using 3 primary categories: molecular function and biological process. The gene ontology analysis provided the overview of functional interpretation of the resultant differential protein expression. In this study, three general terms were used to classified the differential dataset as biological process, molecular function, and cellular component. In the GO molecular function category, the upregulated proteins in comparisons between the CCDS group and both the ageing group and the adult group were similarly classified into 4 groups: binding, molecular function regulator, catalytic activity and transporter activity. In the GO biological process category, the upregulated proteins in comparisons between the CCDS group and both the ageing group and the adult group were similarly classified into 4 groups: metabolic process, cellular process, biological regulation and transporter activity (Fig. 5).

Pathway enrichment analysis of CCDS proteins of interest using STRING showed some relation between amyloid precursor protein and some proteins of interest. Proteins at the core of the traffic link have good protein-protein interactions.

From 87 of proteins that matched, among which 48 and 41 proteins showed at least 1.5-fold differences in their expression levels according to the emPAI values in the CCDS vs ageing and CCDS vs adult group in comparison. The downregulated proteins in comparisons of the CCDS group with both the adult group and the ageing group included 4 proteins: alpha-2-macroglobulin, alpha-1B-glycoprotein, complement factor B and immunoglobulin lambda-like polypeptide 5-like. The downregulated proteins, involved in blood coagulation and the complement cascade. The upregulated proteins in the comparisons of the CCDS group with both the ageing group and the adult group were specifically involved in several biological processes. The biological process of upregulated proteins linked to neurodegenerative disease was mostly blood coagulation, acute phase protein and complement cascade, as shown in Table 2.

**Table 2:** Downregulated and upregulated proteins in comparisons of the CCDS group with both the adult and the ageing

Accession number^**a**^	Protein name	Protein mass	pI	Protein score	Biological process	***P***-value
**Downregulated proteins**						
gi|345792424	alpha-2-macroglobulin	165114	6.27	101	negative regulation of complement activation	*p* = 0.0144
gi|545487024	alpha-1B-glycoprotein	61261	5.81	47	platelet degranulation	*p* = 0.0001
gi|345778397	complement factor B	86266	7.18	75	regulation of complement activation	*p* = 0.1544
gi|545544683	immunoglobulin lambda-like polypeptide 5-like	24739	6.41	1116	innate immune response	*p* = 0.0049
**Upregulated proteins**						
gi|73955106	apolipoprotein A-I	30163	5.28	2244	lipoprotein metabolic process	*p* = 0.0136
gi|704000372	apolipoprotein A-IV	42510	5.75	318	removal of superoxide radicals	*p* < 0.0001
gi|345799905	predict apolipoprotein A-IV	43795	5.34	615	removal of superoxide radicals	*p* < 0.0001
gi|545488191	apolipoprotein E isoform X5	47029	8.45	88	regulation of amyloid beta clearance	N/A
gi|73978329	fibrinogen alpha chain	96583	5.76	275	blood coagulation	*p* = 0.002
gi|73977992	fibrinogen gamma chain isoformX1	49286	5.74	1092	blood coagulation	*p* < 0.0001
gi|120141	fibrinogen gamma chain, partial	2688	4.55	93	blood coagulation	*p* = 0.201
gi|57109938	kininogen-1	48317	5.58	104	blood coagulation	*p* < 0.0001
gi|545485785	plasminogen isoformX1	90952	6.75	121	blood coagulation	*p* = 0.1496
gi|130314	plasminogen	36654	8.48	152	blood coagulation	*p* < 0.0001
gi|123511	haptoglobin	36434	5.72	2272	acute phase response	*p* = 0.0001
gi|545560457	inter-alpha-trypsin inhibitor heavy chain H4 isoformX1	113355	7.1	292	acute phase response	*p* < 0.0001
gi|359321961	prothrombin	70259	5.71	42	acute phase response and blood coagulation	N/A
gi|345803075	C4b-binding protein alpha chain isoform X1	68505	7.77	171	complement activation classical pathway	*p* < 0.0001
gi|50979240	clusterin precursor	51757	5.65	107	complement activation and regulation of Aβ formation	*p* < 0.0001
gi|598107	IgA heavy chain constant region	37255	6.06	114	complement activation classical pathway	*p* < 0.0001
gi|19715661	Ig J chain	12733	4.94	38	innate immune response	*p* = 0.0789
gi|73995687	Ig lambda-like polypeptide 5-like	14832	8.84	1528	complement activation classical pathway	*p* < 0.0001
gi|345777714	alpha-1-acid glycoprotein 1 isoform X1	23291	5.38	45	regulation of immune response	*p* = 0.0245
gi|545531456	plasma protease C1 inhibitor	48,128	5.51	88	complement activation classical pathway	*p* < 0.0001
gi|50978658	alpha-fetoprotein precursor	68738	5.77	52	cellular protein metabolic process	*p* = 0.027
gi|256574824	glutathione peroxidase 3 precursor	25363	8.79	59	response to oxidative stress	*p* < 0.0001
gi|44888810	hemoglobin alpha chain	15208	7.98	267	cellular oxidant detoxification	*p* < 0.0001
gi|73988725	hemopexin	51305	6.88	149	heme metabolic process	*p* < 0.0001
gi|119637837	pigment epithelium-derived factor	44236	8.69	40	aging	*p* < 0.0001
gi|57089193	transthyretin isoform 2	15858	6.42	619	retinol, thyroid hormone transport	*p* = 0.001

^a^ Accession number from NCBInr database for *Canis* spp.

N/A = cannot measure by ANOVA because the samples all have a standard error of zero.

**Fig. 6:**
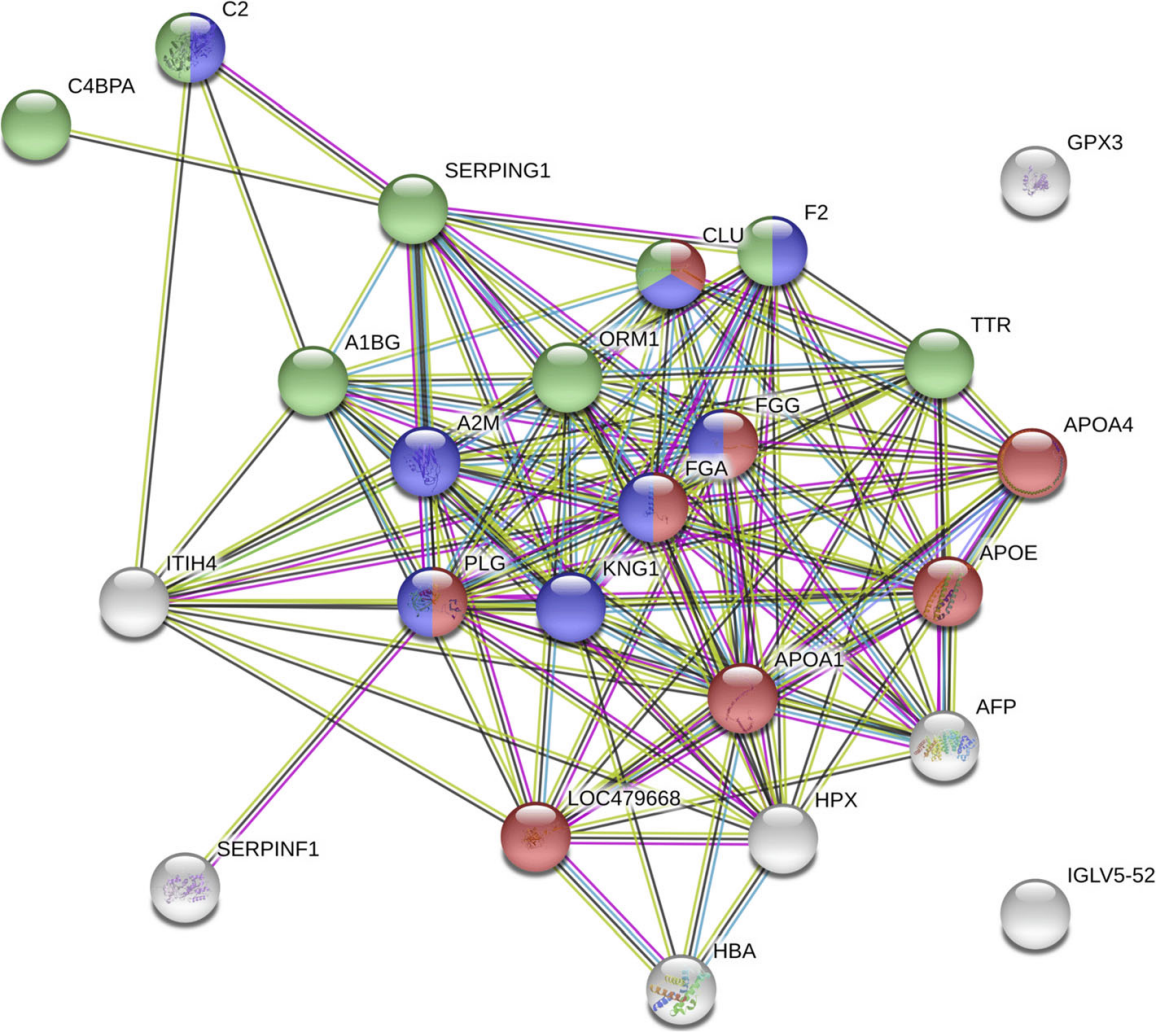
STRINGS protein-protein interaction: Analysis of protein changes in the CCDS group compared with both the adult group and the ageing group (total proteins = 24, red colour = response to stress, blue colour = complement and coagulation cascades and green = innate immune system)

The downregulated and upregulated proteins which reported in the Table 2 showed protein score higher than 95% in confidence. The differential expression was shown from the semi-quantification by selecting the altered proteins with at least two replicates. To explore the potential proteins, we performed a pathway analysis by using STRING version 11.0. Total protein changes in comparisons of the CCDS group with both the ageing group and the adult group were expanded to show the evidence of an interaction, giving a total of 24 proteins. We compared this protein set to those of the Gene Ontology, Kyoto Encyclopedia of Genes and Genomes (KEGG) and Reactome pathways databases. Most proteins involved with the Gene Ontology biological process category are stress response proteins. Most proteins involved with the KEGG and Reactome pathway databases are complement and coagulation cascade and immune system proteins, respectively (Fig. 6).

## Discussion

Currently, CCDS can be diagnosed by using a screening questionnaire, but no biomarkers have been identified. In our study, the plasma Aβ_42_ level did not show a correlation with the questionnaire score and could not distinguish CCDS dogs from normal dogs. Our experiment indicated that the plasma Aβ_42_ level in ageing group had a higher level compared to an adult group which shown a similarity to the previous study of Aβ level in brain of canine that showed the increasing of oligomeric Aβ’s accumulation with age [4, 40]. In AD studies, results for the use of plasma Aβ_42_ as a biomarker have been controversial [34]. The expression was in contrast with that reported in a previous study, which showed that plasma Aβ_42_ was significantly gradually decreased in early CCDS and severe CCDS, while the highest Aβ_42_ plasma level was observed in younger dogs [7, 33]. Some studies in human and rat models found increased Aβ_42_ or cleavage at the onset of AD; conversely, in the later stage, Aβ_42_ levels were decreased in the CSF and plasma, which may be caused by plaque deposition [41–43]. Next, we performed nano-LC-MS/MS to provide a dataset of potential biomarkers to improve the diagnosis of CCDS. In our study, plasma Aβ_42_ could not be detected and identified by nano-LC-MS/MS, which may be because of abundant protein interference, including that from immunoglobulin and albumin [44].

Using the raw MS/MS data, a biological heat map analysis of CCDS, ageing and adult dogs was performed. The results indicated the differentiation of the 3 sample groups according to the clustered pattern of their expression. In the enrichment analysis, differentially expressed plasma proteins were involved in complement and coagulation cascades or were acute phase proteins or apolipoproteins. This finding suggested that CCDS was enhanced by the increase in inflammation in peripheral organs, leading to the activation of the acute phase response and complement and coagulation cascades that partly functioned by apolipoproteins. Nano-LC-MS/MS analysis was used to discover the underlying mechanisms of CCDS.

Aβ can trigger inflammation and activate the complement cascade classical and alternative pathways [45, 46]. Complement downstream induced a proteolytic cascade, resulting in the opsonization of Aβ from the brain to the peripheral circulation. Complement component 4 binding protein binds to Aβ_42_ in the brain and is elevated in the plasma and CSF of AD samples [47]. Aβ plays a role not only in inflammation but also in the coagulation cascade. There is an association between haemostatic factors and inflammatory mechanisms in AD [36]. In our study, we found an increase in plasminogen, fibrinogen and kininogen. In AD, plasminogen was found to colocalize with Aβ plaques [36], while fibrinogen was capable of enhancing Aβ aggregation and fibrillization, causing impairment in AD [48, 49]. Patients with higher levels of plasma fibrinogen and plasminogen modulating neuroinflammation had worsening cognitive decline and Aβ deposition [36, 50, 51].

There is an interaction between the acute phase response proteins that arise in early inflammation and other inflammatory pathways. The acute phase response is part of the innate immune system that responds to systemic inflammation. In our study, we found an increase in acute phase proteins (haptoglobin and prothrombin). The increase in acute phase proteins is generally related to defence against physiological damage and the restoration of homeostasis. Haptoglobin can bind misfolded proteins to prevent Aβ aggregation [52]. Moreover, prothrombin, localized within the vascular endothelium, was upregulated to shrink at microvascular sites [53]. Our proteomic results were in accordance with human AD studies, and the comparison showed increased haptoglobin and prothrombin in the plasma of AD patients, indicating an increased risk for cognitive decline and deterioration [54, 55]. The downregulation of alpha-1B-glycoprotein in CCDS dogs was present as in the AD study; however, the exact mechanism of alpha-1B-glycoprotein is not yet known [56].

Apo E and apolipoprotein A-I (apo A-I), the major apolipoproteins present in CSF, influence neurodegeneration via cholesterol and lipid metabolism [57, 58]. Apo A-I or apo E can bind with cholesterol to form high-density lipoprotein (HDL)-like particles that are important for neurons in membrane growth and repair [59]. Apo E can be measured in both CSF and blood; however, the use of apo E as a potential biomarker in AD is inconsistent and controversial [60]. In our study, apo E, apo A-I and apo A-IV were increased in the CCDS group. In accordance with other studies, apo E colocalizes with capillary Aβ in the brains of aged dogs and humans [61]. Interestingly, the human *APOE ε4* gene has been reported as a major genetic risk factor for late-onset AD [62]. In AD studies, apo A-I has the capability to prevent the formation of Aβ_42_ and reduce Aβ_42_ toxicity, and immunohistochemistry revealed the colocalization of apo A-I with Aβ_42_ [63].

The plasma Aβ_42_ level was lower in the CCDS group, which may be due to an increase in clearance mechanisms. Our results show a high level of sequester proteins or Aβ binding proteins due to the clearance mechanism. The clearance of Aβ occurs by binding with soluble Aβ to prevent aggregation and increase degrading mechanisms [20]. Many studies have suggested that alpha-2-macroglobulin, apolipoproteins, transthyretin, clusterin and the complement system are involved in AD pathogenesis through the sequestration of Aβ, leading to increased Aβ clearance in vivo [64–67]. Transthyretin and clusterin are sequester proteins that function as inhibitors of Aβ fibril formation and further suppress the toxicity of oligomers. A previous study in human and transgenic mouse models indicated that the plasma clusterin concentration was significantly increased in AD patients and was associated with the level of fibrillar Aβ in the brain. Moreover, plasma transthyretin levels were also significantly increased in comparisons between patients with AD and controls [35, 68, 69]. Aβ sequester proteins may have a dual function by reducing the formation of toxic species and increasing clearance and degradation through LRP-1-mediated endocytosis [20]. However, another study showed no statistically significant expression of serum fibrinogen, lipoprotein A and plasminogen-activator-inhibitor-1 in AD patients [70]. Therefore, this set of sequester proteins needs more study for use as a blood-based biomarker of CCDS.

This study reports a proteomic finding on CCDS from the point of view of protein expression. However, there were some limitations. The main limitation of the present study was limit number of dogs. Our study used a pooling sera from both healthy and diseased dogs which reduced the sample size. On the other hand, by this pooling sample technique also eliminates the estimation of inter-individual variation within each group. However, this method could result in biomarker loss and reduce the applicability of the biomarker upon validation. Another limitation of this study was only using the quantification of biomarkers from plasma. Ideally, biomarkers for evaluate CCDS should be incorporate both CSF and plasma.

## Conclusions

Blood biomarkers have the potential to be used as diagnostic tools for evaluating CCDS. Our study revealed evidence for the existence of a specific blood-based proteomics profile of CCDS from domestic dogs in Thailand, which may be an interesting tool for diagnostic purposes. Plasma Aβ_42_ detection may be insufficient to distinguish CCDS dogs from normal ageing dogs. Our present findings suggest the predictive underlying mechanisms of cognitive dysfunction syndrome in dogs: the co-occurrence of inflammation-mediated acute phase response proteins and complement and coagulation cascades that partly function by apolipoproteins. Some of the differentially expressed proteins need validation to serve as potential predictor biomarkers along with the use of a questionnaire for improved CCDS diagnosis. Further study, our proteomic results provide a list of potential biomarkers that require validation by other techniques for assessing the progression of cognitive decline. A study of the association between plasma biomarker panels and core pathological features of CCDS is also needed.

## Methods

### Patient enrolment

Experiments on Thai domestic dogs were carried out at the Prasu-Arthorn Animal Hospital, Faculty of Veterinary Science, Mahidol University. Client-owned dogs were recruited: 8 ageing dogs (age > 7 years), 4 adult dogs (age range 1–7 years) and 10 dogs that were diagnosed with CCDS. The exclusion criteria were brain diseases other than CCDS or concurrent medical problems that mimic signs of cognitive impairment. CCDS was classified according to the CCDR questionnaire rating score [27]. The study protocol was approved by the Mahidol University Animal Care and Use Committee (AICUC) (UI-01287-2558).

### Blood sampling

Blood samples were collected from the cephalic or jugular vein into vials containing ethylenediaminetetraacetic acid (EDTA), and the samples were centrifuged at 3000 rpm for 10 min. Plasma was divided into 2 aliquots and kept at − 80 °C. The first aliquot was used for the ELISA procedure, and the second aliquot was used for the proteomics study.

### ELISA for Aβ_42_ detection

Plasma Aβ_42_ of all dogs in each group was quantified using specific sandwich ELISA kits for humans (Elabscience, Wuhan, China) in accordance with the manufacturer’s instructions as described. Briefly, plates were incubated with 100 μL of sample or standard for 90 min at 37 °C. The liquid was then removed from each well. Biotinylated antibody was added to the plates and incubated for 1 h at 37 °C. After several wash steps, 100 μL horseradish peroxidase (HRP)-conjugated working solution was added to each well and incubated for 30 min at 37 °C. After repeated wash steps, the substrate solution was then added. Positive samples developed a blue colour. The reaction was stopped by the addition of stop solution and further measured at 450 nm.

### Sodium dodecyl sulfate-polyacrylamide gel electrophoresis (SDS-PAGE)

Prior to gel-based separation, each plasma was pooled in the same volume and diluted at 1:1 ratio with 1% Triton X-100, 1% NaCl and 1% SDS. The samples were measured protein concentration by Bradford assay (Bio-Rad, Benicia, CA, USA) at 590 nm with bovine serum albumin (Thermo Fisher, Waltham, MA, USA) as a standard. For gel-based separation by SDS-PAGE, total of 30 μg of protein was used to load into each lane of 12% SDS-PAGE. After that, the gel was stained with Coomassie Brilliant Blue R-250 (Bio-Rad, Benicia, CA, USA) and de-stained with 30% ethanol (Merck, Darmstadt, Germany) in 10% acetic acid (Merck, Darmstadt, Germany). The gel was then scanned using a GS-710 scanner (Bio-Rad, Benicia, CA, USA). The protein band was divided into 11 segments per lane according to size and chopped into 1 mm^3^ pieces. For protein identification, each piece was subjected to in-gel digested prior to being subjected to nano liquid chromatography tandem mass spectrometry (nano-LC-MS/MS).

### In-gel digestion

Gel pieces were de-stained using 50% acetonitrile (ACN) (Thermo Fisher, Waltham, MA, USA) in 50 mM ammonium bicarbonate (Merck, Darmstadt, Germany). After that, disulfide bonds were reduced with 4 mM dithiothreitol (DTT) (Omnipur, Darmstadt, Germany) in 50 mM ammonium bicarbonate for 10 min at 60 °C. Gel pieces were alkylated in 250 mM iodoacetamide (IAA) in 50 mM ammonium bicarbonate for 30 min at room temperature in the dark. The gel pieces were dehydrated 2 times in 100% ACN for 15 min and dried at room temperature. Then, trypsin (Sigma-Aldrich, St Louis. MO, USA) in 50 mM ammonium bicarbonate was added, and the gel pieces were incubated overnight at 37 °C. The tryptic peptides were extracted from the gels using 100% ACN. Finally, peptide mixtures were dried in a vacuum centrifuge to dryness and kept at − 80 °C until further nano-LC-MS/MS analysis.

### Analysis of peptide patterns by nano-LC-MS/MS

After tryptic digestion, all peptide solution was completely dried and reconstituted in 15 μL of 0.1% formic acid (Merck, Darmstadt, Germany). Five μL of each sample were subjected to the nano-LC/MS-MS three times. Peptide separation was performed on a C18 column. The flow rate was set at 300 nL/min. The elution occurred during the 30-min gradient from the 4% mobile phase B (80% acetonitrile in 0.1% formic acid) to the 50% mobile phase A (0.1% formic acid in water), and the eluent was infused into a microTOF-Q (Bruker Daltonics, Bremen, Germany). The mass spectra from the mass spectrometry (MS) and tandem mass spectrometry (MS/MS) covered mass ranges of m/z 400–2000 and m/z 50–1500, respectively.

### LC-MS/MS data analysis

**Fig. 7:**
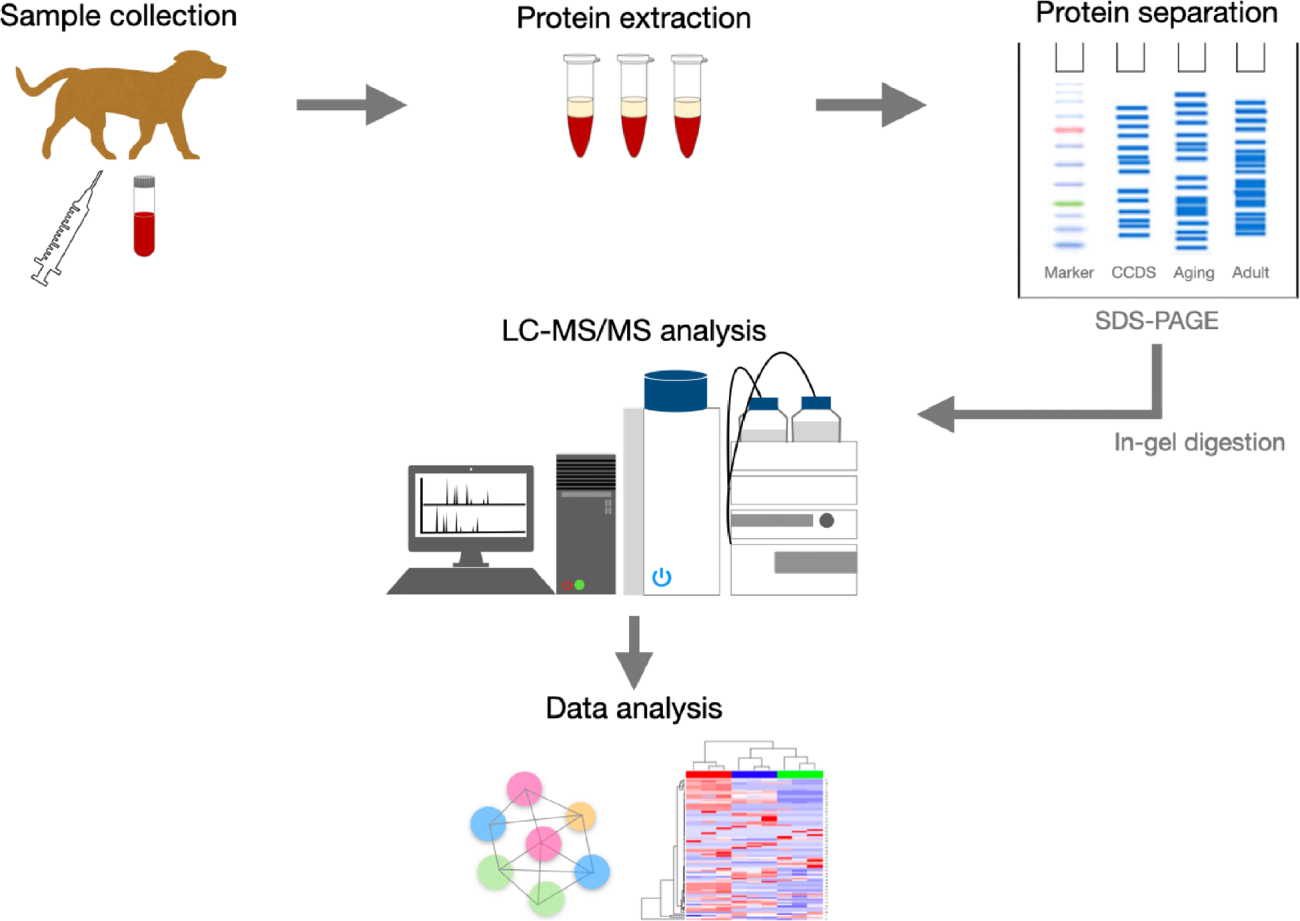
Proteomics workflow (The copyright belongs to the authors)

The mass spectrometric analysis was performed by data-dependent acquisition. None of exclusion condition was used. The electrospray voltage was 4500 V and collision energy was 10 eV. LC-MS/MS data files were converted to a mascot generic file (.mgf) format with DataAnalysis 3.4 version software. Mascot daemon version 2.3.02 (Matrix Science, London, UK) was used to merge the .mgf files and to identify the proteins against those in the NCBInr database (24 October 2019). The entries was 61,390,244 sequences. Mammalia was set as the taxonomy filter. Missed cleavage was set to 1, the peptide tolerance was set to 0.8 Da, and the tandem MS tolerance was set to 0.6 Da. Variable modifications were set to include methionine oxidation and cysteine carbamidomethylation. Selecting protein hits were with *p*-value ≤ 0.05. The proteins that reported in this research were 95% confidence. The protein expression was quantified by peptide count analysis using the emPAI value provided by Mascot. Differentially expressed proteins in at least two of the biological replicates were reported as protein alterations in each group. Processed protein-level data were analysed through a range of software tools. A heat map was constructed using the R studio program. Protein-protein interaction network and functional analysis, based on GO enrichment, KEGG, and Reactome pathways, were analysed using online STRING software (https://www.string-db.org) at the default setting. The graphic of the proteomic workflow is shown in Fig. 7.

### Statistical analysis

Statistical analysis was performed using descriptive statistical procedures and software (GraphPad Prism, Version 5). Pearson correlation analyses were used to examine the correlations between the CCDR score and plasma Aβ_42_ levels. The statistical significance of differences in the sex was determined by chi-square test. The statistical significance of differences in the haematological parameters and plasma Aβ_42_ levels between groups was determined with the Kruskal-Wallis test, followed by a Dunn’s Multiple Comparison test (*p*-value < 0.05 was considered statistically significant). Statistical analysis of the proteomic data was performed using one-way ANOVA on each protein to evaluate the *p*-value between different groups (*p*-value < 0.05 was considered statistically significant).

## Supplementary Information

**Additional file 1 Table S2.** Downregulated and upregulated proteins in comparisons of the CCDS with both the adult and the ageing.

**Additional file 2.** Fold change of downregulated and upregulated proteins in comparisons of the CCDS with both adult and ageing (supplement information).

**Additional file 3.** Protein identification from differential proteomics experiment (supplement information).

**Additional file 4.** Original uncropped gel (supplement information).

## Data Availability

The mass spectrometry proteomics data have been deposited to the ProteomeXchange Consortium via the PRIDE partner repository (https://www.ebi.ac.uk/pride/) with the dataset identifier PXD023301**.**

## Abbreviations

1D-SDS-PAGE: One-dimensional sodium dodecyl sulfate-polyacrylamide gel electrophoresis
Aβ_42_: Amyloid beta 42
AD: Alzheimer’s disease
ACN: Acetonitrile
ALT: Alanine aminotransferase
apo A-I: Apolipoprotein A-I
apo A-IV: Apolipoprotein A-IV
apo E: Apolipoprotein E
CCDR: Canine Cognitive Dysfunction Rating scale
CCDS: Canine cognitive dysfunction syndrome
DTT: Dithiothreitol
EDTA: Ethylenediaminetetraacetic acid
emPAI: Exponentially Modified Protein Abundance Index
GO: Gene Ontology
HCT: Haematochrit
IAA: Iodoacetamide
KEGG: Kyoto Encyclopedia of Genes and Genomes
LC: Liquid chromatography
LRP1: Lipoprotein receptor-related protein 1
mgf: Mascot generic file
MS/MS: Tandem mass spectrometry
NCBInr: Non-redundant National Center Biotechnology Information
WBC: White blood cells
