# Supplementary material for: Change in the plasma proteome associated with canine cognitive dysfunction syndrome (CCDS) in Thailand
Source: BMC Vet Res. 2021 Jan 29;17:60. doi: 10.1186/s12917-021-02744-w (PMC7845120; doi:10.1186/s12917-021-02744-w)
Supplement: Supplementary file 1 — Additional file 1 Table S2. Downregulated and upregulated proteins in comparisons of the CCDS with both the adult and the ageing. [file 12917_2021_2744_MOESM1_ESM.docx]

**Table 2.** Downregulated and upregulated proteins in comparisons of the CCDS group with both the adult and the ageing

| **Downregulated proteins** | | | | | | |
| --- | --- | --- | --- | --- | --- | --- |
| **Accession number^a^** | **Protein name** | **Protein mass** | **pI** | **Protein score** | **Biological process** | ***P*-value** |
| gi\|345792424 | alpha-2-macroglobulin | 165114 | 6.27 | 101 | [negative regulation of complement activation](https://www.ebi.ac.uk/QuickGO/term/GO:0001869) | *p* = 0.0144 |
| gi\|545487024 | alpha-1B-glycoprotein | 61261 | 5.81 | 47 | [platelet degranulation](https://www.ebi.ac.uk/QuickGO/term/GO:0002576) | *p* = 0.0001 |
| gi\|345778397 | complement factor B | 86266 | 7.18 | 75 | [regulation of complement activation](https://www.ebi.ac.uk/QuickGO/term/GO:0030449) | *p* = 0.1544 |
| gi\|545544683 | immunoglobulin lambda-like  polypeptide 5-like | 24739 | 6.41 | 1116 | [innate immune response](https://www.ebi.ac.uk/QuickGO/term/GO:0045087) | *p* = 0.0049 |
| **Upregulated proteins** | | | | | | |
| **Accession number^a^** | **Protein name** | **Protein mass** | **pI** | **Protein score** | **Biological process** | ***P*-value** |
| gi\|73955106 | apolipoprotein A-I | 30163 | 5.28 | 2244 | lipoprotein metabolic process | *p* = 0.0136 |
| gi\|704000372 | apolipoprotein A-IV | 42510 | 5.75 | 318 | removal of superoxide radicals | *p* < 0.0001 |
| gi\|345799905 | predict apolipoprotein A-IV | 43795 | 5.34 | 615 | removal of superoxide radicals | *p* < 0.0001 |
| gi\|545488191 | apolipoprotein E isoform X5 | 47029 | 8.45 | 88 | regulation of amyloid beta clearance | N/A |
| gi\|73978329 | fibrinogen alpha chain | 96583 | 5.76 | 275 | blood coagulation | *p* = 0.002 |
| gi\|73977992 | fibrinogen gamma chain isoformX1 | 49286 | 5.74 | 1092 | blood coagulation | *p* < 0.0001 |
| gi\|120141 | fibrinogen gamma chain, partial | 2688 | 4.55 | 93 | blood coagulation | *p* = 0.201 |

^a^ Accession number from NCBInr database for *Canis* spp.

N/A = cannot measure by ANOVA because the samples all have a standard error of zero

**Table 2.** Downregulated and upregulated proteins in comparisons of the CCDS group with both the adult and the ageing (cont.)

| **Accession number^a^** | **Protein** **name** | **Protein mass** | **pI** | **Protein score** | **Biological process** | ***P*-value** |
| --- | --- | --- | --- | --- | --- | --- |
| gi\|57109938 | kininogen-1 | 48317 | 5.58 | 104 | blood coagulation | *p* < 0.0001 |
| gi\|545485785 | plasminogen isoformX1 | 90952 | 6.75 | 121 | blood coagulation | *p* = 0.1496 |
| gi\|130314 | plasminogen | 36654 | 8.48 | 152 | blood coagulation | *p* < 0.0001 |
| gi\|123511 | haptoglobin | 36434 | 5.72 | 2272 | acute phase response | *p* = 0.0001 |
| gi\|545560457 | inter-alpha-trypsin inhibitor heavy  chain H4 isoformX1 | 113355 | 7.1 | 292 | acute phase response | *p* < 0.0001 |
| gi\|359321961 | prothrombin | 70259 | 5.71 | 42 | acute phase response and blood coagulation | N/A |
| gi\|345803075 | C4b-binding protein alpha chain isoform X1 | 68505 | 7.77 | 171 | complement activation classical pathway | *p* < 0.0001 |
| gi\|50979240 | clusterin precursor | 51757 | 5.65 | 107 | complement activation and regulation of Aβ formation | *p* < 0.0001 |
| gi\|598107 | IgA heavy chain constant region | 37255 | 6.06 | 114 | complement activation classical pathway | *p* < 0.0001 |
| gi\|19715661 | Ig J chain | 12733 | 4.94 | 38 | innate immune response | *p* = 0.0789 |
| gi\|73995687 | Ig lambda-like polypeptide 5-like | 14832 | 8.84 | 1528 | complement activation classical pathway | *p* < 0.0001 |
| gi\|345777714 | alpha-1-acid glycoprotein 1 isoform X1 | 23291 | 5.38 | 45 | regulation of immune response | *p* = 0.0245 |
| gi\|545531456 | plasma protease C1 inhibitor | 48128 | 5.51 | 88 | complement activation classical pathway | *p* < 0.0001 |

^a^ Accession number from NCBInr database for *Canis* spp.

N/A = cannot measure by ANOVA because the samples all have a standard error of zero

**Table 2.** Downregulated and upregulated proteins in comparisons of the CCDS group with both the adult and the ageing (cont.)

| **Accession number^a^** | **Protein** **name** | **Protein mass** | **pI** | **Protein score** | **Biological process** | ***P*-value** |
| --- | --- | --- | --- | --- | --- | --- |
| gi\|50978658 | alpha-fetoprotein precursor | 68738 | 5.77 | 52 | [cellular protein metabolic process](https://www.ebi.ac.uk/QuickGO/term/GO:0044267) | *p* = 0.027 |
| gi\|256574824 | glutathione peroxidase 3 precursor | 25363 | 8.79 | 59 | response to oxidative stress | *p* < 0.0001 |
| gi\|44888810 | hemoglobin alpha chain | 15208 | 7.98 | 267 | cellular oxidant detoxification | *p* < 0.0001 |
| gi\|73988725 | hemopexin | 51305 | 6.88 | 149 | heme metabolic process | *p* < 0.0001 |
| gi\|119637837 | pigment epithelium-derived factor | 44236 | 8.69 | 40 | aging | *p* < 0.0001 |
| gi\|57089193 | transthyretin isoform 2 | 15858 | 6.42 | 619 | retinol, thyroid hormone transport | *p* = 0.001 |

^a^ Accession number from NCBInr database for *Canis* spp.

N/A = cannot measure by ANOVA because the samples all have a standard error of zero
