# Supplementary material for: Change in the plasma proteome associated with canine cognitive dysfunction syndrome (CCDS) in Thailand
Source: BMC Vet Res. 2021 Jan 29;17:60. doi: 10.1186/s12917-021-02744-w (PMC7845120; doi:10.1186/s12917-021-02744-w)
Supplement: Supplementary file 2 — Additional file 2. Fold change of downregulated and upregulated proteins in comparisons of the CCDS with both adult and ageing (supplement information). [file 12917_2021_2744_MOESM2_ESM.docx]

**Supplement Table.** Fold change of downregulated and upregulated proteins in comparisons of the CCDS with both adult and ageing

| **Accession number^a^** | **Protein** **name** | **Fold change (CCDS compare ageing group)** | **Fold change (CCDS compare adult group)** |
| --- | --- | --- | --- |
| gi\|704000372 | RecName: Full=Apolipoprotein A-IV; Short=Apo-AIV; Short=ApoA-IV; AltName: Full=Apolipoprotein A4; Flags: Precursor | 22.2500 | Found only in CCDS |
| gi\|345799905 | PREDICTED: apolipoprotein A-IV | 20.3750 | Found only in CCDS |
| gi\|57109938 | PREDICTED: kininogen-1 isoformX2 | 5.8571 | 5.8571 |
| gi\|73978329 | PREDICTED: fibrinogen alpha chain | 5.3571 | 10.7143 |
| gi\|123511 | RecName: Full=Haptoglobin; AltName: Full=Zonulin; Contains: RecName: Full=Haptoglobin alpha chain; Contains: RecName: Full=Haptoglobin beta chain | 4.8043 | 14.6271 |
| gi\|545560457 | PREDICTED: inter-alpha-trypsin inhibitor heavy chain H4 isoform X1 | 4.7778 | Found only in CCDS |
| gi\|545485785 | PREDICTED: plasminogen isoform X1 | 4.7500 | Found only in CCDS |
| gi\|120141 | RecName: Full=Fibrinogen gamma chain, partial | 3.4452 | Found only in CCDS |
| gi\|73977992 | PREDICTED: fibrinogen gamma chain isoformX1 | 3.2059 | 2.7595 |
| gi\|73988725 | PREDICTED: hemopexin | 3.0000 | 3.0000 |
| gi\|57089193 | PREDICTED: transthyretin isoform 2 | 2.4921 | 3.7087 |
| gi\|73955106 | PREDICTED: apolipoprotein A-I | 2.300113 | 3.6011 |
| gi\|598107 | IgA heavy chain constant region | 2.2632 | Found only in CCDS |
| gi\|345803075 | PREDICTED: C4b-binding protein alpha chain isoform X1 | 2.2000 | 4.4000 |
| gi\|545531456 | PREDICTED: plasma protease C1 inhibitor | 2.1429 | Found only in CCDS |
| gi\|55742764 | serum albumin precursor | Found only in CCDS | No difference |
| gi\|73995687 | PREDICTED: immunoglobulin lambda-like polypeptide 5-like | Found only in CCDS | Found only in CCDS |
| gi\|44888810 | RecName: Full=Hemoglobin subunit alpha; AltName: Full=Alpha-globin; AltName: Full=Hemoglobin alpha chain | Found only in CCDS | Found only in CCDS |
| gi\|130314 | RecName: Full=Plasminogen; Contains: RecName: Full=Plasmin heavy chain A; Contains: RecName: Full=Plasmin light chain B, partial | Found only in CCDS | 6.0000 |

**Supplement Table.** Fold change of downregulated and upregulated proteins in comparisons of the CCDS with both adult and ageing (con’t)

| **Accession number^a^** | **Protein** **name** | **Fold change (CCDS compare ageing group)** | **Fold change (CCDS compare adult group)** |
| --- | --- | --- | --- |
| gi\|256574824 | glutathione peroxidase 3 precursor | Found only in CCDS | Found only in CCDS |
| gi\|19715661 | immunoglobulin J chain | Found only in CCDS | Found only in CCDS |
| gi\|545505255 | PREDICTED: CD5 molecule-like | Found only in CCDS | No difference |
| gi\|345777714 | PREDICTED: alpha-1-acid glycoprotein 1 isoform X1 | Found only in CCDS | Found only in CCDS |
| gi\|50979240 | clusterin precursor | Found only in CCDS | Found only in CCDS |
| gi\|119637837 | pigment epithelium-derived factor | Found only in CCDS | Found only in CCDS |
| gi\|545488191 | PREDICTED: apolipoprotein E isoform X5 | Found only in CCDS | Found only in CCDS |
| gi\|73967363 | PREDICTED: alpha-2-antiplasmin isoformX2 | Found only in CCDS | No difference |
| gi\|50978658 | alpha-fetoprotein precursor | Found only in CCDS | Found only in CCDS |
| gi\|359321961 | PREDICTED: prothrombin | Found only in CCDS | Found only in CCDS |
| gi\|73977990 | PREDICTED: fibrinogen beta chain isoformX2 | No difference | 7.3333 |
| gi\|545556445 | PREDICTED: fibronectin, partial | No difference | 7.2500 |
| gi\|74003556 | PREDICTED: fetuin-B | No difference | 3.125 |
| gi\|121583756 | serpin peptidase inhibitor, clade A (alpha-1 antiproteinase, antitrypsin), member 1 precursor | No difference | 2.8696 |
| gi\|73975215 | PREDICTED: vitamin D-binding protein isoformX2 | No difference | 2.846154 |
| gi\|73995681 | PREDICTED: immunoglobulin lambda-like polypeptide 5-like | No difference | 2.59396 |
| gi\|50979230 | apolipoprotein C-III precursor | No difference | 2.4375 |
| gi\|124390009 | immunoglobulin heavy chain constant region CH2 | No difference | 1.828125 |
| gi\|17066526 | immunoglobulin gamma heavy chain B | No difference | 1.828125 |
| gi\|545539001 | PREDICTED: LOW QUALITY PROTEIN: serotransferrin isoform 1 | No difference | 1.59009 |
| gi\|54792721 | beta-2-glycoprotein 1 precursor | No difference | Found only in CCDS |
| gi\|73975797 | PREDICTED: serum paraoxonase/arylesterase 1 isoform 2 | No difference | Found only in CCDS |
| gi\|73990367 | PREDICTED: ceruloplasmin isoformX1 | No difference | Found only in CCDS |
| gi\|545497726 | PREDICTED: RPA-interacting protein isoform X1 | No difference | Found only in CCDS |
| gi\|61740600 | keratin, type I cytoskeletal 10 | No difference | Found only in CCDS |

**Supplement Table.** Fold change of downregulated and upregulated proteins in comparisons of the CCDS with both adult and ageing (con’t)

| **Accession number^a^** | **Protein** **name** | **Fold change (CCDS compare ageing group)** | **Fold change (CCDS compare adult group)** |
| --- | --- | --- | --- |
| gi\|545487024 | PREDICTED: alpha-1B-glycoprotein | -5.2000 | -3.6000 |
| gi\|345792424 | PREDICTED: alpha-2-macroglobulin isoform X2 | -2.5 | -3.6667 |
| gi\|74005944 | PREDICTED: complement factor H isoform 2 | -2.3889 | 3.6000 |
| gi\|73998292 | PREDICTED: retinol-binding protein 4 | -2.1818 | Found only in CCDS |
| gi\|545520262 | PREDICTED: complement C4-A | -2.0909 | 4.3333 |
| gi\|17066530 | immunoglobulin gamma heavy chain D | -2.027778 | No difference |
| gi\|545535669 | PREDICTED: complement C3 | -1.775 | 1.809524 |
| gi\|345778397 | PREDICTED: complement factor B | -1.625 | -2.6250 |
| gi\|124390013 | immunoglobulin heavy chain constant region CH4 | No difference | -2.4167 |
| gi\|74003532 | PREDICTED: prolyl 3-hydroxylase 2 isoform 1 | Found only in ageing | No difference |
| gi\|545544683 | PREDICTED: immunoglobulin lambda-like polypeptide 5-like isoform X2 | Found only in ageing | Found only in adult |
| gi\|73975156 | PREDICTED: immunoglobulin J chain isoform 3 | Found only in ageing | No difference |
| gi\|73981174 | PREDICTED: T-cell surface antigen CD2 | Found only in ageing | No difference |
| gi\|545518174 | PREDICTED: gelsolin | Found only in ageing | No difference |
| gi\|359321423 | PREDICTED: piggyBac transposable element-derived protein 2-like isoform X1 | Found only in ageing | No difference |
| gi\|545534209 | PREDICTED: LOW QUALITY PROTEIN: tyrosine-protein kinase JAK3 | Found only in ageing | No difference |
| gi\|545525368 | PREDICTED: maltase-glucoamylase, intestinal | Found only in ageing | No difference |
| gi\|57036446 | PREDICTED: apolipoprotein E isoformX1 | No difference | Found only in adult |
| gi\|545544650 | PREDICTED: uncharacterized protein LOC100855594 | No difference | Found only in adult |

^a^ Accession number from NCBInr database for *Canis* spp.
