# Supplementary figures and images for: Change in the plasma proteome associated with canine cognitive dysfunction syndrome (CCDS) in Thailand
Source: BMC Vet Res. 2021 Jan 29;17:60. doi: 10.1186/s12917-021-02744-w (PMC7845120; doi:10.1186/s12917-021-02744-w)

Marker

CCDS

Ageing

Adult

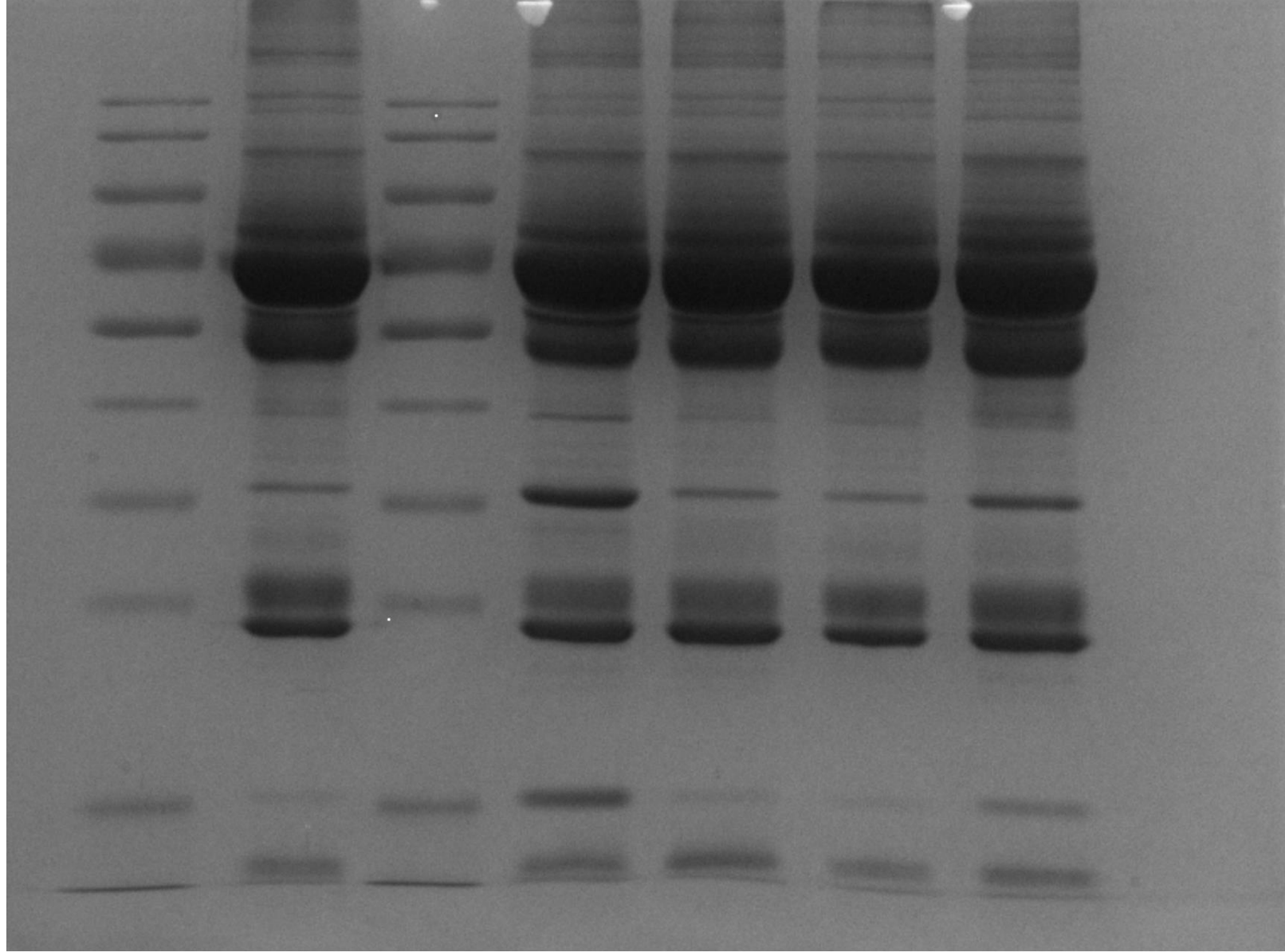

Supplement: Supplementary file 4 — Additional file 4. Original uncropped gel (supplement information). [file 12917_2021_2744_MOESM4_ESM.pdf]
